# The Resistance Landscape of Uropathogens in a Romanian Tertiary Center: A 13-Month Single-Center Study with Focus on *Klebsiella* and Urease-Positive Organisms

**DOI:** 10.3390/microorganisms14071491

**Published:** 2026-07-08

**Authors:** Oana Nicu-Canareica, Loredana Sabina Cornelia Manolescu, Maria Luiza Băean, Cosmin Medar, Cristian Nicu, Gabriela-Cristiana Ivan, Ștefana-Georgiana Tanasă, Theodor-Georgian Badea, Georgian-Florentin Nedelea, Alexandru Gabriel Berza, Vlad-Octavian Bolocan, Maria Glencora Costache, Gelu-Adrian Popa, Viorel Jinga

**Affiliations:** 1 Doctoral School, Faculty of Medicine, Carol Davila University of Medicine and Pharmacy, 020021 Bucharest, Romania; oana.canareica@drd.umfcd.ro (O.N.-C.);; 2 Department of Fundamental Sciences, Faculty of Midwifery and Nursing, University of Medicine and Pharmacy “Carol Davila”, 050474 Bucharest, Romania; loredana.manolescu@umfcd.ro (L.S.C.M.); maria.costache@umfcd.ro (M.G.C.); geluadrianpopa@yahoo.com (G.-A.P.); 3 Clinical Laboratory of Medical Microbiology, Marius Nasta Institute of Pneumology, 050159 Bucharest, Romania; 4 Department of Clinical Laboratory of Radiology and Medical Imaging, CF2 Clinical Hospital, 011464 Bucharest, Romaniatheodor.badea@umfcd.ro (T.-G.B.); 5 Department of Clinical Laboratory of Radiology and Medical Imaging, Clinical Hospital “Prof. Dr. Th. Burghele”, 050664 Bucharest, Romania; 6 Department of Laboratory Medicine, “Prof. Dr. Th. Burghele”, 050659 Bucharest, Romania; 7 Department of Laboratory of Radiology and Medical Imaging, Sf. Ioan Clinical Emergency Hospital, 042122 Bucharest, Romania; 8 Department of Urology, Clinical Hospital “Prof. Dr. Th. Burghele”, Faculty of Medicine, University of Medicine and Pharmacy “Carol Davila”, 050474 Bucharest, Romania; viorel.jinga@umfcd.ro; 9 Medical Sciences Section, Academy of Romanian Scientists, 050085 Bucharest, Romania

**Keywords:** antimicrobial resistance, urinary tract infection, uropathogens, *Klebsiella pneumoniae*, multidrug resistance, CRE, urease-positive bacteria, Romania

## Abstract

Urinary tract infections (UTIs) sit at a leading edge of the antimicrobial-resistance (AMR) crisis, and Romania ranks among the most affected European countries, yet large single-center series of urinary isolates with complete antibiograms remain scarce. We describe the full resistance landscape of urinary pathogens at a Romanian tertiary hospital, with particular attention to multidrug-resistant *Klebsiella* and to the often-overlooked urease-positive organisms. We analyzed all positive urine cultures processed over 13 consecutive months (February 2025–February 2026). The first isolate per patient was the primary unit of analysis (*n* = 2331); the full isolate-level dataset (*n* = 3348) was analyzed secondarily. Resistance phenotypes (putative ESBL, carbapenem resistance, MDR, VRE) were derived from per-agent susceptibility data following Magiorakos et al., and resistance rates were calculated only on tested isolates. Associations were tested with chi-square/Fisher tests and Benjamini–Hochberg correction. A total of 3348 isolates from 2331 unique patients were analyzed (median age 68 years; 55% male). *Escherichia coli* predominated (40.5%), followed by *Enterococcus* spp. (22.2%) and *Klebsiella* spp. (18.9%). Among the first isolates, 15.3% were MDR; 16.7% of *E. coli*, *Klebsiella* and *Proteus* showed a putative ESBL phenotype; and 3.9% of *Enterobacterales* were carbapenem-resistant. Resistance was concentrated in *Klebsiella* (36.2% MDR, 22.8% putative ESBL, 14.7% carbapenem-resistant; all higher than *E. coli*, *p* < 0.001) and in *Pseudomonas* spp. (48.1% carbapenem-resistant). *E. coli* retained excellent activity to nitrofurantoin (1.2%), fosfomycin (1.2%; tested in a subset) and ertapenem (0.1%) despite high trimethoprim–sulfamethoxazole (31.5%) and fluoroquinolone (24.3%) resistance. Urease-positive organisms formed a distinct subgroup with high trimethoprim–sulfamethoxazole resistance but preserved carbapenem activity. The urinary resistance burden was substantial but uneven, concentrated in *Klebsiella* and *Pseudomonas.* These findings support a stratified, locally guided empirical strategy and combined stewardship–infection control efforts.

## 1. Introduction

Urinary tract infections (UTIs) are among the most common bacterial infections worldwide, with an estimated 404.6 million incident cases and around 5.2 million disability-adjusted life-years globally in 2019 [1]. Because uropathogens are predominantly Gram-negative and evolutionarily adapted to acquire both virulence and resistance determinants, UTIs sit at the leading edge of the antimicrobial resistance (AMR) crisis [2]. In a dedicated Global Burden of Disease analysis, *Escherichia coli* and *Klebsiella pneumoniae* together accounted for more than half of UTI deaths attributable to and associated with bacterial AMR. The two single deadliest pathogen–drug combinations were third-generation cephalosporin-resistant and fluoroquinolone-resistant *E. coli* [1]. In 2024, the World Health Organization reaffirmed this hierarchy, listing carbapenem-resistant *K. pneumoniae* and third-generation-cephalosporin-resistant *E. coli* as its top two critical-priority pathogens [3,4]. Surveillance of uropathogen resistance is therefore a key protective activity.

The clinical importance of UTI surveillance extends beyond uncomplicated cystitis. Complicated UTIs (cUTIs), encompassing acute pyelonephritis and catheter-associated UTIs (CAUTIs), carry substantially greater morbidity, higher rates of empirical treatment failure, and inpatient mortality rates reported between 0% and 50% depending on the population studied [5]. These cases concentrate the AMR burden disproportionately: in a recent rehabilitation centre cohort, carbapenem resistance reached 51.4% in *Pseudomonas aeruginosa* and 37.5% in *K. pneumoniae*, and MDR infection was independently associated with prolonged hospital stay [6]. Hospitalized populations of older adults with multiple comorbidities and indwelling devices therefore deserve particular attention.

AMR is not distributed evenly across Europe. Successive ECDC/EARS-Net reports show that resistance levels are consistently highest in the southern, central and eastern parts of the continent [4]. The trend is worsening: the estimated EU incidence of carbapenem-resistant *K. pneumoniae* bloodstream infections rose by 61% between 2019 and 2024 [7]. Within this gradient, the 2024 ECDC Surveillance Atlas ranked Romania the third most affected European country for carbapenem-resistant K. pneumoniae from invasive infections (50.3%), behind only Greece (60.2%) and Bulgaria (67.6%) [4]. This is precisely why local Romanian data carry weight: they describe a setting at the steep end of the European resistance distribution, where empirical therapy assumptions imported from Western guidelines are least likely to hold.

Despite Romania’s prominence on the European resistance map, granular local evidence remains scarce. National EARS-Net reporting captures invasive isolates and resistance percentages but not resistance mechanisms or enzyme types, and there is no national surveillance system dedicated to carbapenemase-producing *Enterobacterales* [4]. Large single-center series of urinary isolates with complete S/I/R antibiograms—the data most relevant to everyday empirical prescribing—are particularly underrepresented [8].

Recent Romania single-center series have documented this pattern across multiple regions: post-pandemic *E. coli* showed rising fluoroquinolone and third-generation cephalosporin resistance in Constanța, while nitrofurantoin and fosfomycin retained activity [9]. A cross-sectional Timișoara analysis confirmed that inpatient uropathogens displayed significantly higher resistance than outpatient isolates across multiple agents [8]; and a male-only cohort from a Bucharest tertiary urology centre reported carbapenem resistance exceeding 20% in Gram-negative isolates overall and *Klebsiella* resistance to both imipenem and meropenem exceeding 30% [10]. These series, however, are typically narrow in population (single sex, single comorbidity, or single organism) or limited to short observation windows. This leaves a need for comprehensive multi-organism series covering the full spectrum of urinary isolates from a defined tertiary catchment.

One subgroup is routinely overlooked: the urease-positive organisms, chiefly *Proteus* spp., *Morganella morganii* and *Providencia* spp. These bacteria hydrolyze urea to ammonia, raising urinary pH and driving the precipitation of magnesium–ammonium–phosphate (struvite) and carbonate–apatite crystals [11]. The resulting crystalline biofilms shelter bacteria from antibiotics and the immune system, block catheters, and seed infection stones [12]. Up to roughly 75% of staghorn (infection) stones are struvite/carbonate–apatite, and urease-producing organisms are the proximate cause [13]. Because these organisms predominate in complicated, catheter-associated and recurrent UTI, their resistance profiles have outsized clinical importance yet are rarely reported as a coherent group.

A second consideration shaping the clinical relevance of urinary AMR data is the patient population. Older adults with diabetes mellitus, indwelling catheters, and other comorbidities are both at elevated risk of UTI and more likely to carry resistant isolates [14]. Stratification by comorbidity has direct empirical consequences: in a recent multi-center analysis, MDR *E. coli* rose from 30.5% in uncomplicated UTI to 61.8% in complicated UTI without diabetes and remained at 59.0% in complicated UTI with diabetes. Patients with diabetes also showed significantly higher nitrofurantoin resistance compared with non-diabetic complicated UTI (34.3% vs. 10.2%, *p* < 0.001) [15]. A recent Timișoara series of 318 patients, from a tertiary hospital, identified advanced age as the primary independent driver of MDR, with risk increasing linearly with each additional year, alongside indwelling catheterization as a strong cofactor [16]. A tertiary-hospital catchment such as ours, which concentrates exactly this population, is therefore the appropriate setting in which to characterize the resistance landscape that local empirical guidance must address.

Granular local data remain particularly scarce: few Romanian single-center series report resistance per patient—the first-isolate estimates most relevant to empirical prescribing—and even fewer do so across the full range of urinary pathogens, including urease-positive and non-fermentative organisms.

Against this background, we conducted an ambispective single-center study of all positive urine cultures processed at a Romanian tertiary hospital over 13 consecutive months. Our objectives were as follows: (i) to describe the species distribution of urinary isolates; (ii) to quantify the overall and per-organism burden of clinically important resistance phenotypes; (iii) to report agent-level resistance for the principal uropathogens, with denominators restricted to tested isolates; and (iv) to characterize the urease-positive subgroup as a distinct entity.

We hypothesized that MDR and carbapenem resistance would be concentrated in *Klebsiella* spp. rather than distributed evenly. We further hypothesized that *E. coli* would retain activity to nitrofurantoin and fosfomycin despite high fluoroquinolone and trimethoprim–sulfamethoxazole resistance; and that the urease-positive group would show a distinct, complicated UTI-type profile.

## 2. Materials and Methods

### 2.1. Study Design and Setting

We conducted a single-center, observational study at a highly representative tertiary urology center in Bucharest, Romania, the Prof. Dr. Th. Burghele Clinical Hospital. All urine specimens processed by the clinical bacteriology laboratory over a 13-month period, from February 2025 through February 2026, were screened for inclusion. This window was chosen as a complete, consecutive set of monthly datasets; data for March 2026 were available but incomplete at the time of extraction and were excluded a priori to preserve a clean analytic period.

The study was designed as a descriptive cross-sectional surveillance series. No comparator cohort or matched control group was defined, since the primary objective was to characterize the species distribution and resistance landscape of all consecutive urinary isolates rather than to test causal associations between exposures and clinical outcomes. Comparisons reported in the Results section are between bacterial subgroups within the cohort and are interpreted as descriptive associations.

### 2.2. Population and Inclusion/Exclusion Criteria

Eligible records were positive urine cultures with a clinically significant bacterial count (typically ≥10^5^ CFU/mL, i.e., >100,000 CFU/mL, per laboratory reporting practice) and a corresponding antibiogram. Specimens reported as sterile, contaminated, or below the significance threshold and non-urinary specimens were excluded. After applying these criteria, the analytic dataset comprised 3348 isolates from 2331 unique patients.

### 2.3. Microbial Identification and Susceptibility Testing

Bacterial identification was performed using conventional manual biochemical methods. Antimicrobial susceptibility testing was carried out by disk diffusion (Oxoid, Basingstoke, UK), and results were interpreted according to Clinical and Laboratory Standards Institute (CLSI) M100 performance standards ed. 35 (2025) [17] and ed. 36 (2026) [18].

Each isolate was categorized per antibiotic as susceptible (S), intermediate (I), resistant (R), or not tested (NT). Susceptible dose-dependent (SDD) results were classified as intermediate. For every resistance rate reported, the denominator is the number of isolates actually tested against that agent; NT and blank results were excluded from both numerator and denominator, so per-drug denominators vary by organism. Antimicrobial testing followed the laboratory’s cascade-reporting policy: ertapenem was the first-line carbapenem tested on all *Enterobacterales*, whereas meropenem, imipenem, fosfomycin and the salvage agents ceftazidime–avibactam and imipenem–relebactam were tested only on isolates already resistant to first-line agents. Denominators for these reserve agents are therefore small, and their resistance percentages reflect a pre-selected subset rather than the whole population.

### 2.4. Resistance Phenotype Definitions

Multidrug-resistant (MDR), extensively drug-resistant (XDR), and pandrug-resistant (PDR) phenotypes were defined according to Magiorakos et al. [13]. MDR was defined as acquired resistance to at least one agent in three or more antimicrobial classes. Comprehensive susceptibility testing across all antimicrobial categories was not available for most isolates under the laboratory’s cascade-reporting policy. Formal XDR and PDR classification was not applied; instead, the most extensively resistant isolates are described individually in the Results section. Extended-spectrum β-lactamases (ESBLs) are, by the widely used working definition, acquired β-lactamases that hydrolyze penicillins and first- to third-generation (oxyimino-) cephalosporins and aztreonam—but not cephamycins or carbapenems—and are inhibited by β-lactamase inhibitors such as clavulanic acid [19].

Because no confirmatory clavulanate synergy test or molecular characterization was available, a putative ESBL phenotype was inferred operationally. It was applied only to *Escherichia coli*, *Klebsiella* spp. and *Proteus mirabilis*—the organisms for which CLSI provides ESBL screening criteria—and defined as acquired resistance to at least one extended-spectrum cephalosporin. The extended-spectrum cephalosporin assessment used ceftazidime, ceftriaxone and cefotaxime, together with amoxicillin–clavulanate, piperacilin–tazobactam and trimethoprim–sulfamethoxazole; testing more than one cephalosporin improves the sensitivity of ESBL detection. This phenotype cannot distinguish ESBL production from AmpC overexpression or other mechanisms and is therefore reported as putative.

Carbapenemases are β-lactamases that additionally hydrolyze carbapenems and span Ambler classes A (e.g., KPC), B (metallo-β-lactamases such as NDM, VIM, IMP) and D (OXA-48-type) [20]. A CRE phenotype was inferred from acquired resistance to at least one carbapenem (ertapenem, meropenem or imipenem).

Ertapenem was the primary carbapenem tested on all *Enterobacterales*. Per CLSI M100 [17,18], ertapenem non-susceptibility is the most sensitive phenotypic indicator of carbapenemase production. However, it can also arise from porin loss combined with ESBL and AmpC activity, and some carbapenemase producers (e.g., OXA-48-type) may test susceptible to expanded-spectrum cephalosporins. Because these assignments are phenotypic, they identify resistance behaviour rather than a specific enzyme, and may incorporate non-enzymatic mechanisms (e.g., porin loss with AmpC hyperproduction); this limitation is addressed in the Discussion section. VRE was defined as vancomycin-resistant *Enterococcus*. Phenotype flags were derived programmatically from the per-agent S/I/R values rather than entered manually.

### 2.5. Handling of Multiple Isolates and Duplicates

**Figure 1:**
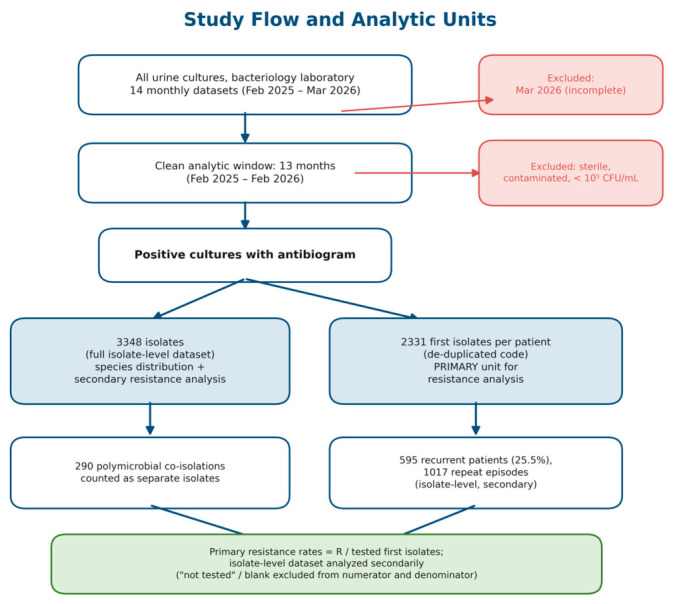
Study flow and analytic units. The isolate is the primary unit for species and resistance; unique patients form the denominator for demographics and recurrence. Red arrows indicate excluded records; navy arrows indicate the analytic flow.

The first isolate per patient was the primary unit of analysis. For each unique patient, identified by a de-duplicated anonymized code, the earliest positive urine culture was retained (*n* = 2331). All primary resistance estimates and between-organism comparisons were computed on these independent first isolates, in line with CLSI M39 [21] recommendations for cumulative susceptibility reporting. The full isolate-level dataset (*n* = 3348) comprised every positive urine culture with its own antibiogram including repeat cultures from the same patient at separate visits and was analyzed secondarily to describe the laboratory workload; it is presented in the Supplementary Materials. Polymicrobial cultures (two organisms from the same specimen) were recorded as two separate isolates; 290 such co-isolation events were present (Figure 1). The risk of selection bias arising from recurrent infections is addressed in the Discussion section.

### 2.6. Statistical Analysis

**Figure 2:**
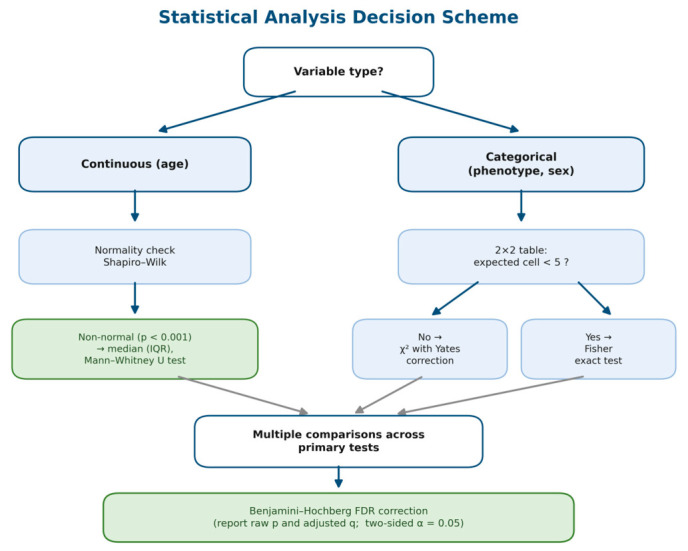
Statistical analysis decision scheme, from variable type through choice of test to multiplicity correction.

Categorical variables are summarized as counts and percentages. Because the age distribution departed significantly from normality (Shapiro–Wilk W = 0.940, *p* < 0.001), continuous age is reported as median with interquartile range (IQR) and compared using the Mann–Whitney U test. Resistance rates were calculated per agent as the proportion of resistant isolates among those tested; isolates recorded as not tested or blank were excluded from both numerator and denominator. Associations between categorical variables were tested with the Pearson chi-square test of independence, with the Fisher exact test substituted whenever any expected cell count was below five. Effect sizes for the principal between-organism comparisons are reported as odds ratios (Ors) with 95% Wald confidence intervals (e.g., *Klebsiella* versus *Escherichia coli* for MDR, ESBL and CRE phenotypes, and *Proteus* versus *E. coli* for trimethoprim-sulfamethoxazole resistance). The Haldane–Anscombe 0.5 correction was applied when any cell count was zero. Temporal trends in monthly CRE positivity among *Klebsiella* isolates were tested with the Cochran–Armitage test for trend and corroborated by logistic regression with month as a continuous predictor. To address the lack of first-isolate-per-patient de-duplication, a pre-specified sensitivity analysis restricted to the first isolate per unique patient (*n* = 2331) was performed; results are reported alongside the isolate-level estimates. All tests were two-sided, and *p* < 0.05 was considered significant. To account for multiple comparisons, *p*-values were adjusted using the Benjamini–Hochberg false-discovery-rate procedure. The analysis was planned as descriptive and hypothesis-generating; no multivariable modelling was undertaken. Analyses were performed in jamovi (version 2.5; The jamovi project, Sydney, Australia), and were independently reproduced from the source dataset using a documented script with identical results (Figure 2).

### 2.7. Ethics

This single-center study used an ambispective design (prospective informed consent at admission; retrospective assembly of the microbiological dataset). It was conducted in accordance with the Declaration of Helsinki and approved by the Institutional Ethics Committee of Prof. Dr. Th. Burghele Clinical Hospital (approval no. 10307/29.10.2024). All patients provided written informed consent at hospital admission, and the microbiological dataset was extracted and analyzed in anonymized form, without personal identifiers.

## 3. Results

### 3.1. Cohort Characteristics

During the 13-month study window, 3348 positive urine-culture isolates were processed from 2331 unique patients. The population was older and male-predominant: median age was 68 years (IQR 56–74; mean 64.1 ± 14.9), and 55.0% of patients were male. Recurrence was common—595 patients (25.5%) had more than one positive culture, contributing 1017 episodes beyond the index isolate. Most recurrent patients had two episodes (*n* = 374), but the distribution had a long tail, including a single patient with ten separate positive cultures.

### 3.2. Species Distribution

**Figure 3:**
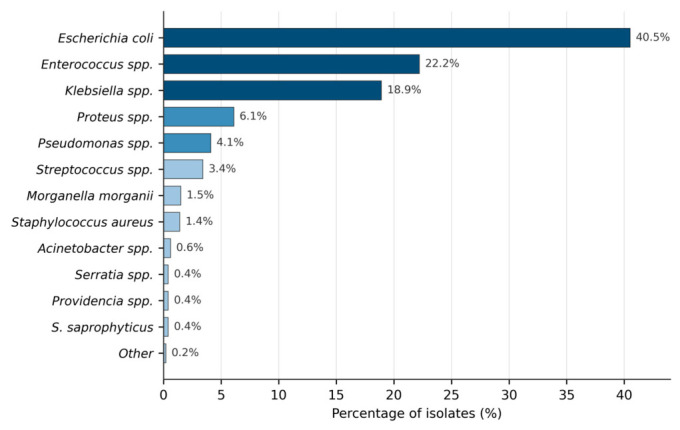
Species distribution of urinary isolates (*n* = 3348). The three dominant organisms account for over 80% of all isolates.

Three organisms accounted for over 80% of all isolates: *Escherichia coli* (40.5%), *Enterococcus* spp. (22.2%) and *Klebsiella* spp. (18.9%). The urease-positive group—*Proteus* spp., *Morganella morganii* and *Providencia* spp.—together represented 7.6% of isolates (Table 1, Figure 3). Species frequencies are reported for all isolates (*n* = 3348); all resistance analyses below use the first isolate per patient (*n* = 2331).

### 3.3. Overall Burden of Resistance Phenotypes

**Figure 4:**
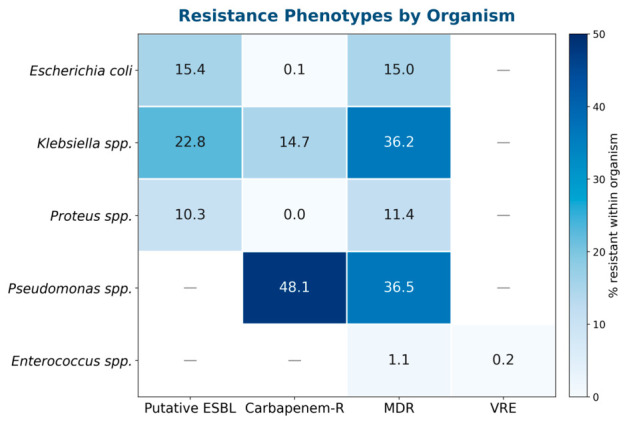
Heatmap of resistance phenotypes by organism (% resistant within each organism). Resistance is concentrated in *Klebsiella* spp. and *Pseudomonas* spp. “—” denotes not applicable.

Among first isolates, 15.3% met MDR criteria. A putative ESBL phenotype was identified in 16.7% of *E. coli*, *Klebsiella* and *Proteus*, and carbapenem resistance in 3.9% of *Enterobacterales*; VRE was rare (a single isolate, 0.2% of enterococci tested). Formal XDR and PDR were not classified (Methods) (Table 2). This burden was highly uneven and concentrated in *Klebsiella* spp. and *Pseudomonas* spp. (Table 3, Figure 4). MDR and ESBL were markedly higher in *Klebsiella* than in *E. coli* (MDR 36.2% vs. 15%, OR 3.21, 95% CI 2.46–4.19; putative ESBL 22.8% vs. 15.4%, OR 1.62, 95% CI 1.22–2.16; both *p* < 0.001). CRE was also far more frequent in *Klebsiella* than in all other *Enterobacterales* combined (14.7% vs. 0.5%, OR 35.05, 95% CI 14.99–81.94; *p* < 0.001). Carbapenem resistance was also strikingly elevated in *Pseudomonas* spp. relative to *Enterobacterales* (48.1% vs. 3.9%, OR 22.55, 95% CI 12.39–41.04; *p* < 0.001).

### 3.4. Resistance in Escherichia coli

**Figure 5:**
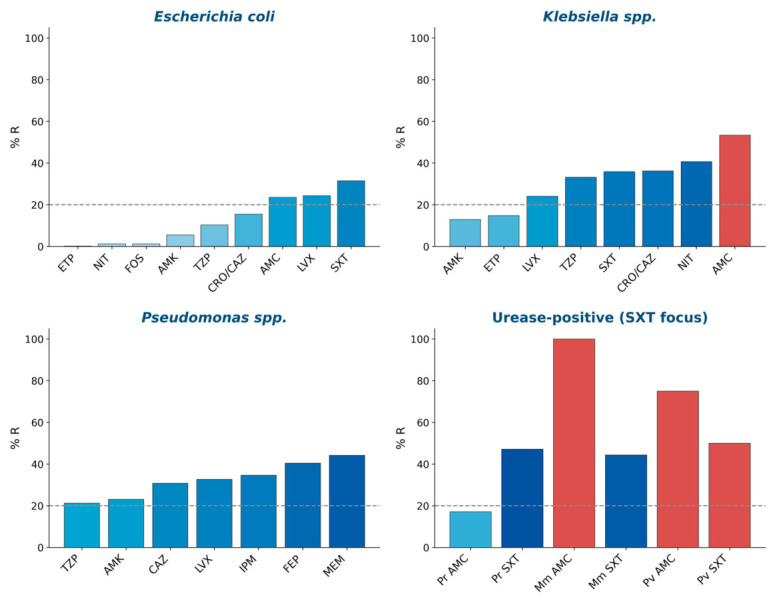
Antimicrobial resistance profiles of the principal uropathogens (% resistant among tested isolates). Dashed line marks the 20% empirical-use threshold. Pr, *Proteus*; Mm, *Morganella morganii*; Pv, *Providencia*.

*E. coli* retained excellent activity for the agents central to uncomplicated UTI: resistance was 1.2% to nitrofurantoin, 1.2% to fosfomycin and 0.1% to ertapenem (Table 4; Figure 5). By contrast, the common oral options were eroded—31.5% resistance to trimethoprim–sulfamethoxazole and 24.3% to levofloxacin. Amoxicillin–clavulanate resistance was 23.5%, while third-generation cephalosporin resistance (15.5%) mirrored the putative ESBL rate. Amikacin remained reliable (5.5%). Fosfomycin was tested in only a minority of isolates (163), so its low resistance must be read with that caveat.

### 3.5. Klebsiella spp. as the MDR/ESBL Hotspot

*Klebsiella* carried by far the heaviest resistance burden among *Enterobacterales*: 36.2% MDR, 22.8% putative ESBL, and 14.7% carbapenem resistance (Table 5). Resistance was high across most agents—amoxicillin–clavulanate 53.4%, third-generation cephalosporins 36.2%, nitrofurantoin 40.7% and trimethoprim–sulfamethoxazole 35.9%. Decisively for empirical therapy, ertapenem resistance—the carbapenem tested on all isolates—reached 14.8%. Meropenem and imipenem were tested only on already-resistant isolates (16/17 and 17/19), a pre-selected escalation subset that does not represent population resistance.

A small subset of first isolates approached the limit of conventionally available therapy. A total of 91 first isolates were carbapenem (*Klebsiella* spp., *n* = 58; *Pseudomonas* spp., *n* = 25; with isolated *Serratia*, *Acinetobacter*, *Enterobacter*, *Providencia*, *Morganella* and *Escherichia coli*). Among these, the reserve β-lactam/β-lactamase-inhibitor combinations were active only variably: ceftazidime–avibactam was tested in 62 and resistant in 45, and imipenem–relebactam tested in 33 and resistant in 31. In total, 51 first isolates were resistant both to carbapenems and to at least one reserve agent tested (*Klebsiella* spp., *n* = 36; *Pseudomonas* spp., *n* = 9; with single *Serratia*, *Acinetobacter*, *Providencia* and *Morganella* isolates), and 25 were resistant to both ceftazidime–avibactam and imipenem–relebactam where both were tested. These difficult-to-treat isolates, concentrated in *Klebsiella* spp. and *Pseudomonas* spp., are described here individually rather than under a formal XDR/PDR rubric. Because colistin and other last-resort agents were not routinely tested under the cascade-reporting policy, a pandrug-resistant designation cannot be assigned, and these counts should be read against the reserve-agent denominators shown.

### 3.6. Enterococci and VRE

*Enterococcus* spp. remained largely susceptible to the agents anchoring enterococcal UTI therapy: 3.5% resistance to ampicillin, 3.1% to nitrofurantoin and 0.7% to fosfomycin, with linezolid (0.2%) and vancomycin (0.2%) essentially preserved (Table 6). A single vancomycin-resistant Enterococcus was identified (0.2% of enterococci tested); the case was manually verified and showed a resistance profile consistent with vancomycin-resistant *Enterococcus faecium* (resistant to ampicillin, penicillin and levofloxacin, susceptible to linezolid). Because enterococci were reported at genus level, the clinically important distinction between *Enterococcus faecalis* and the more resistance-prone *Enterococcus faecium* could not be made; this is acknowledged as a limitation. Fluoroquinolone resistance was higher (levofloxacin 27.4%), limiting their empirical use.

### 3.7. The Urease-Positive Subgroup

The urease-positive organisms displayed distinct profiles (Table 7). *Proteus* spp. (*n* = 123) remained carbapenem-susceptible (ertapenem 0.0%) and largely spared on amikacin (1.6%) and piperacillin–tazobactam (4.1%), yet trimethoprim–sulfamethoxazole resistance was high at 47.1%—significantly higher than in *E. coli* (31.5; OR 1.93, 95% CI 1.32–2.83; *p* < 0.001); nitrofurantoin and colistin are intrinsically inactive against *Proteus*, *Morganella* and *Providencia* and were therefore not interpreted or reported for these genera [17,18]. *Morganella morganii* (*n* = 27) was uniformly resistant to amoxicillin–clavulanate (100%, intrinsic) with trimethoprim–sulfamethoxazole at 44.4%, but carbapenems retained (ertapenem 3.7%). *Providencia* spp. (*n* = 4) followed the same pattern; with only four first isolates these estimates are unstable and are shown for completeness only. Together these genera behave as a coherent complicated-UTI subset rather than community pathogens.

### 3.8. Pseudomonas spp. and Temporal Variation

*Pseudomonas* spp. (*n* = 52) added a non-fermenter dimension to the carbapenem resistance burden: 48.1% were carbapenem-resistant and 36.5% were MDR. Resistance was 44.2% to meropenem, 40.4% to cefepime, and 32.7% to levofloxacin; the least-affected agents tested were piperacillin–tazobactam (21.2%) and amikacin (23.1%) (Table 8; Figure 5).

**Figure 6:**
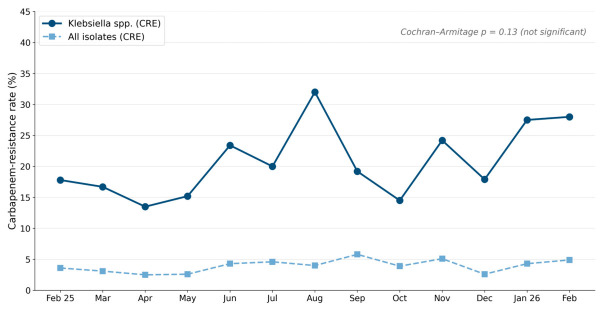
Monthly CRE rate across the study period. *Klebsiella* spp. CRE showed substantial month-to-month variability (13.5–32.0%). A formal trend test (Cochran–Armitage Z = 1.53, *p* = 0.13; logistic regression OR per month 1.046, 95% CI 0.987–1.109, *p* = 0.13) did not support a sustained upward trend, so monthly percentages should be read as descriptive.

Monthly isolate volume ranged from 162 to 362, with no clear seasonal pattern in overall MDR (11.5–21.6%). The CRE rate among *Klebsiella* isolates showed substantial month-to-month variability (range 13.5–32.0%; Table 9; Figure 6), with values descriptively higher toward the end of the study period (27.5% in January 2026, 28.0% in February 2026) than at baseline (17.8% in February 2025). However, a formal test for trend did not support a sustained ascending pattern. The Cochran–Armitage test yielded Z = 1.53 (*p* = 0.13), and logistic regression with month as a continuous predictor produced an odds ratio of 1.046 per month (95% CI 0.987–1.109; *p* = 0.13). Per-month *Klebsiella* denominators are small (25–83 isolates), and these monthly percentages should therefore be read as descriptive rather than as evidence of a sustained upward trend; the variability nevertheless warrants continued surveillance. Across the full period, the carbapenem resistance rate was 19.9% among *Klebsiella* spp. (126/632; 95% CI 17.0–23.2) and 3.9% among all isolates (135/3348; 95% CI 3.4–4.8). The MDR rate was 18% (601/3348; 95% CI 16.7–19.3) (Wilson score intervals).

### 3.9. Secondary Analysis: Full Isolate-Level Dataset

The primary analyses above were restricted to the first isolate per unique patient (*n* = 2331). As a secondary analysis, the principal estimates were recomputed on the full isolate-level dataset (*n* = 3348), which counts every positive culture, including repeats. Because 25.5% of patients contributed multiple cultures (30.4% of all isolates), recurrent—and often more resistant—infections inflate isolate-level estimates relative to the primary first-isolate analysis. The overall species hierarchy and the principal between-organism contrasts were preserved (Table 10). Compared with the primary first-isolate estimates, isolate-level resistance was generally higher, be it modestly for *Escherichia coli* (MDR 15.0% → 16.4%; putative ESBL 15.4% → 17.1%; CRE 0.1% → 0.1%) and *Klebsiella* spp. (MDR 36.2% → 41.1%; putative ESBL 22.8% → 22.6%; CRE 14.7% → 19.9%) or most markedly for *Pseudomonas* spp. carbapenem resistance (48.1% → 51.1%).

This pattern indicates that resistant isolates, particularly *Pseudomonas* spp., originated disproportionately from patients with multiple cultures, so isolate-level rates should be interpreted with this caveat.

The effect-size estimates for the principal between-organism comparisons were robust across both levels. The MDR *Klebsiella* versus *Escherichia coli* odds ratio was 3.21 (95% CI 2.46–4.19) at first-isolate level and 3.57 (2.88–4.42) at isolate level. The CRE contrast for *Klebsiella* versus other *Enterobacterales* remained extreme (OR 35.05 [14.99–81.94] and 45.21 [22.82–89.56], respectively), and the *Pseudomonas* versus *Enterobacterales* carbapenem-resistance contrast was 22.55 (12.39–41.04) and 16.56 (11.35–24.16). All comparisons remained highly significant (*p* < 0.001). The complete isolate-level tables are provided in the Supplementary Materials.

## 4. Discussion

In this single-center series of 3348 urinary isolates from 2331 patients over 13 months, 1 in 7 isolates was multidrug-resistant (15.3%), and the resistance burden was strikingly concentrated rather than diffuse. *E. coli*—the dominant uropathogen at 40.5%—retained excellent activity for the agents central to uncomplicated UTI, whereas *Klebsiella* spp. emerged as the principal reservoir of multidrug and carbapenem resistance. A second non-fermenter focus was seen in *Pseudomonas* spp.

Our findings sit squarely on the steep east-European resistance gradient. ECDC/EARS-Net data for 2024 confirm that AMR levels remain highest in southern, central and eastern Europe [4]. The 2024 Surveillance Atlas ranked Romania third in Europe for carbapenem-resistant *K. pneumoniae* from invasive infections (50.3%), behind Greece (60.2%) and Bulgaria (67.6%) [4]. Our urinary *Klebsiella* CRE rate (14.7%) is lower than these invasive-isolate figures—expected, since urinary isolates include community and colonizing strains—but still far above Western European levels [4].

One recent Romanian tertiary-center urine series provides the closest comparator, with low nitrofurantoin/fosfomycin resistance but high trimethoprim–sulfamethoxazole and rising fluoroquinolone/cephalosporin resistance in *E. coli* [22], a national pattern our data reinforce. Because we lacked syndrome-level clinical data (e.g., cystitis vs. pyelonephritis, catheter status, community vs. hospital onset), our resistance estimates cannot be translated directly into empirical therapy recommendations for individual syndromes that differ in pharmacokinetic requirements.

This caveat is particularly relevant for nitrofurantoin. Although *E. coli* nitrofurantoin resistance was low (1.2% at first-isolate level), nitrofurantoin reaches adequate concentrations only in the lower urinary tract. This finding therefore supports the continued role of nitrofurantoin and fosfomycin for uncomplicated lower-tract infection, but not for upper-tract or systemic infection.

Across other Romanian centers, the same pattern recurs. A five-year *E. coli* analysis from Bucharest reported persistently high ampicillin (48–55.2%), trimethoprim–sulfamethoxazole (22.9–34%) and ciprofloxacin (21.4–31.5%) resistance, with ESBL production peaking at 17.6% during the pandemic [23]. A post-pandemic series of male urology patients from Bucharest found *Klebsiella* resistance to both imipenem and meropenem exceeding 30% [10]. A series of 102 complicated UTI similarly reported *E. coli* (50%) and *Klebsiella* as the dominant pathogens, with an overall MDR rate of 40.2% alongside high penicillin (74.5%), trimethoprim–sulfamethoxazole (58.8%) and fluoroquinolone (49%) resistance [24]. Finally, an Iași cohort of 354 *Enterobacterales* urinary infections documented 24% mortality in DTR or CRE cases versus 1% in fully susceptible cases and identified inappropriate empirical therapy and urinary catheterization as independent predictors of death [25]. Together, these series consistently show that resistance is concentrated in *Klebsiella* rather than *E. coli*, with CRE emergence as the central concern.

The contrast with Western and Northern Europe is sharpest for *Klebsiella* and the CRE phenotype. Whereas the EU incidence of carbapenem-resistant *K. pneumoniae* bloodstream infections rose by 61% between 2019 and 2024, absolute levels in Western/Northern countries remain low. Mechanistically, Eastern European carbapenem-resistant *K. pneumoniae* is increasingly driven by NDM and OXA-48-type (and combined) enzymes, in contrast to the more limited picture of Western isolates [7,26]. This regional enzymology is mirrored in other high-burden settings that have been characterized molecularly. In a study of carbapenem-resistant *Enterobacterales* from urinary samples in India, NDM and OXA-48-like genes co-occurred in 44 of 110 isolates and accounted for nearly all carbapenemase production, with KPC absent [27,28,29,30]. A parallel observation from Nepal identified the blaOXA-48 gene in 42.8% of carbapenemase-producing uropathogens, distributed between *E. coli* and *K. pneumoniae* [29]. An Egyptian uropathogen series likewise identified blaNDM as the dominant carbapenemase gene (37% of CRE) [31].

Comparable patterns appear in other resource-limited settings. A four-year Ghanaian regional series reported *E. coli* (47.8%) and *K. pneumoniae* (15.8%) as dominant uropathogens with 60% of *E. coli* and 25% of *Klebsiella* meeting MDR/ESBL criteria [32]. A Pakistani urinary cohort identified blaCTX-M-15 as the dominant ESBL variant (28.6%) alongside detection of blaOXA-48, blaNDM-1, blaVIM-1 and blaKPC-2 [33].

Two recent Romanian WGS-based studies further clarify the molecular landscape that our phenotypic data describe. In a Bucharest tertiary hospital, Gheorghiță and colleagues recently reported the first identification in Romania of *K. pneumoniae* sequence type ST383 co-harbouring *bla*NDM-5 and *bla*OXA-48. This lineage accounted for 30.7% of all CRE isolates and 51.8% of those carrying both carbapenemase genes; 93.5% of ST383 isolates additionally carrying *bla*CTX-M-15; and the resistance determinants located on mosaic resistance–virulence plasmids and, in part, on the chromosome [34]. Complementing this picture from the virulence side, Lazăr, Nedu and colleagues provided the first detailed molecular description in Romania of hypervirulent *K. pneumoniae*, reporting 15 community-acquired liver abscesses caused by the pandemic ST23-K1 lineage carrying *rmpA*/*rmpA2*, *magA*, *peg-344* and aerobactin-encoding genes on large IncFIB/IncHI1B plasmids. Notably, 93.3% of these isolates were phenotypically wild-type-susceptible. Together, these reports show that the Romanian *Klebsiella* burden has two distinct molecular faces—a healthcare-associated multidrug-resistant pole and a community-acquired hypervirulent pole—each of which demands different surveillance and infection control responses [35].

The convergence on NDM/OXA-48-type enzymes outside the KPC-dominated US setting reinforces the case for local molecular surveillance. Our phenotypic definition cannot distinguish enzyme classes (Figure S1)—which is a limitation—but the high local rate is consistent with this regional enzymology.

The concentration of resistance in *Klebsiella* has direct empirical consequences. With ertapenem resistance at 14.8% and near-universal resistance among the few isolates tested against meropenem, carbapenems can no longer be assumed active against *Klebsiella* in our setting for severe or healthcare-associated UTI. Even amikacin (12.9%) and piperacillin–tazobactam (33.2%) provide incomplete cover.

Our *Klebsiella* burden is consistent with reports from other high-resistance settings. In a Vietnamese five-year serial cross-sectional surveillance, *K. pneumoniae* resistance consistently exceeded 50% for most agents except amikacin, ertapenem, imipenem, meropenem and fosfomycin [36]. In a recent Romanian Iași case–control study, carbapenem-resistant *K. pneumoniae* urinary infections were independently associated with prior healthcare exposure and indwelling devices [37,38].

A follow-up Iași case–control study further refined this picture. It identified neoplasia, neurological disorders, antibiotic exposure in the previous 180 days, and prior carbapenem treatment as independent risk factors for CR-UTI, with *Klebsiella* spp. and *Pseudomonas aeruginosa* accounting for over 90% of CR cases and NDM/KPC as the most frequently identified carbapenemase genes [38]. These data, together with ours, position *Klebsiella* as the principal driver of empirical therapy failure in tertiary urinary care and argue against the reflex use of carbapenems without antibiogram guidance. Empirical regimens for suspected *Klebsiella* UTI should be guided by local antibiograms and, where possible, by rapid resistance mechanism testing rather than by carbapenem reflex.

In sharp contrast, the outlook for uncomplicated *E. coli* UTI is reassuring. Resistance to nitrofurantoin (1.2%) and fosfomycin (1.2%) was minimal, while ertapenem was essentially fully active (0.1%). These agents should remain the empirical backbone for uncomplicated lower UTI, sparing fluoroquinolones (24.3%) and trimethoprim–sulfamethoxazole (31.5%), both of which now exceed the conventional 20% threshold above which empirical use for acute uncomplicated cystitis is discouraged by IDSA/ESCMID guidance [39]. The robustness of nitrofurantoin and fosfomycin against *E. coli* observed here is corroborated by multiple recent series.

A pre-/post-pandemic *E. coli* analysis in Constanța, Romania, reported preserved nitrofurantoin (*p* = 0.26) and fosfomycin (*p* = 0.64) activity despite rising fluoroquinolone and ceftriaxone resistance [9]. A rehabilitation-center cohort similarly documented that *E. coli* retained high susceptibility to nitrofurantoin and fosfomycin against a background of 69.5% ciprofloxacin resistance [6]. A Vietnamese serial cross-sectional study noted 80–90% *E. coli* susceptibility to fosfomycin and the carbapenems [36]. The agreement across geographies supports nitrofurantoin and fosfomycin as durable first-line choices for uncomplicated lower UTI.

The urease-positive subgroup (7.6% of isolates) behaved as a coherent complicated-UTI cluster rather than community pathogens. Their shared features—high trimethoprim-sulfamethoxazole resistance, intrinsic amoxicillin–clavulanate resistance in *Morganella* and *Providencia*, and the intrinsic nitrofurantoin non-susceptibility of the group—narrow empirical options precisely in the catheterized, recurrent, structurally abnormal patients in whom these organisms predominate (Figure S2). Two lines of evidence underpin this clustered behaviour [11,40]. First, the urease-driven alkalinization of urine independently amplifies nitrofurantoin resistance. In a large single-system emergency-department analysis of 67,271 urinalyses, *Proteus* growth was associated with significantly more alkaline urine (OR 2.20, 95% CI 2.06–2.36) and the proportion of nitrofurantoin-susceptible isolates fell from 80.4% at pH 5–7 to 54.6% at pH ≥ 9 [41].

Second, the Morganellaceae carry a characteristic intrinsic resistance signature—chromosomal AmpC β-lactamases, uniform colistin resistance, and an imipenem-non-susceptible/meropenem-and-ertapenem-susceptible carbapenem phenotype—that shapes empirical choice independently of acquired resistance [30]. Both phenomena are consistent with the *Proteus*/*Morganella*/*Providencia* profile we observed and reinforce the rationale for handling these organisms as a coherent complicated UTI subgroup. Pathogenetically, the urease-positive uropathogens combine urolithogenic potential, biofilm formation, and catheter colonization in a way that has long been recognized as central to complicated UTI [11,30] and to catheter-associated UTI in particular [40]. Importantly, carbapenems remained active, preserving a reliable escalation option.

Taken together, these data argue for a stratified, locally guided empirical strategy rather than a one-size-fits-all protocol: narrow-spectrum oral agents for uncomplicated community UTI; early de-escalation away from fluoroquinolones and trimethoprim-sulfamethoxazole; and mechanism-aware, antibiogram-driven choices for *Klebsiella* and *Pseudomonas* in the healthcare-associated setting. The substantial month-to-month variability in *Klebsiella* CRE rates, although not a statistically significant trend, underlines the need for sustained surveillance alongside antibiotic stewardship and infection-control measures.

This study has several limitations. First, it is single-center and, although patients were prospectively consented at admission, the microbiological data were assembled and analyzed retrospectively, which limits generalizability and control over data capture. Second, we lacked individual-level clinical data (ward, catheter status, prior antibiotic exposure, community vs. hospital onset, outcomes), so we could not adjust for case-mix. Third, as a tertiary referral center, our case-mix is likely enriched for complicated and healthcare-associated infections, biasing resistance rates upward. The Romanian CRE landscape is not confined to tertiary academic centers. A single-year analysis from a county emergency hospital in eastern Romania documented 27 carbapenemase-producing urinary isolates with both inter-hospital and intra-hospital transmission across 12 wards and 4 healthcare units in two counties [28], suggesting that the regional burden may extend well beyond academic settings.

Fourth, resistance phenotypes were assigned phenotypically, without molecular confirmation of enzyme class. Phenotypic ESBL and CRE definitions identify resistance behaviour rather than a specific enzyme and may capture non-enzymatic mechanisms such as porin loss combined with AmpC hyperproduction. The reported ESBL and CRE rates should be read as phenotypic estimates.

The magnitude of this phenotype–genotype discordance has been quantified in a recent Egyptian CAUTI series of 132 Gram-negative isolates. Total concordance varied from 80.5% to 91% depending on the resistance database used, with *Pseudomonas* and meropenem showing the greatest discordance [42]. Fifth, the cohort derives from positive cultures rather than from clinically adjudicated infection episodes, so colonization and contamination cannot be fully excluded. The primary analyses were restricted to the first isolate per patient. The full isolate-level dataset, reported as a secondary analysis (Section 3.9), counts recurrent cultures repeatedly (25.5% of patients, 30.4% of isolates) and therefore gave systematically higher rates while preserving the species hierarchy and effect-size estimates, most notably for *Pseudomonas* carbapenem resistance.

Sixth, the study is descriptive by design, without a comparator cohort or matched controls; it therefore characterizes the resistance landscape rather than testing causal hypotheses about individual risk factors for resistance. Case–control designs from other Romanian centers [37,38] complement this descriptive picture by identifying specific predictors of carbapenem-resistant UTI, and the two approaches are best read as complementary.

Seventh, enterococci were characterized at the genus level, so the single vancomycin-resistant isolate could not be assigned to a species. Because vancomycin resistance differs substantially between *Enterococcus faecium* and *E. faecalis*, this VRE finding (one isolate, 0.2% of enterococci tested) should be interpreted cautiously.

Eighth, reserve agents (meropenem, imipenem, ceftazidime–avibactam and imipenem–relebactam) were tested only on isolates already resistant to first-line agents under the cascade-reporting policy. Their resistance percentages derive from small, resistance-enriched denominators and overstate resistance relative to all isolates rather than representing population-wide estimates.

Future work should pair phenotypic surveillance with molecular characterization of carbapenemase genes to clarify the local NDM/OXA-48/KPC balance and guide the choice between newer β-lactam agents. Multi-center and prospective designs, linkage to clinical outcomes, and genomic tracking of the suspected *Klebsiella* cluster would strengthen the conclusions.

## 5. Conclusions

In this 13-month single-center series of 3348 urinary isolates from 2331 patients, the urinary resistance burden was substantial but markedly uneven. Multidrug and carbapenem resistance clustered in *Klebsiella* spp. (36.2% MDR, 22.8% putative ESBL, 14.7% CRE) and, among non-fermenters, in *Pseudomonas* spp. (48.1% carbapenem resistance, 36.5% MDR). In contrast, *E. coli* retained excellent activity to nitrofurantoin (1.2%), fosfomycin (1.2%) and ertapenem (0.1%) even as trimethoprim–sulfamethoxazole (31.5%) and fluoroquinolones (24.3%) crossed the conventional 20% threshold at which empirical use becomes unreliable.

The urease-positive organisms—*Proteus*, *Morganella morganii* and *Providencia*—behaved as a coherent complicated UTI subgroup with high trimethoprim–sulfamethoxazole resistance but preserved carbapenem activity. A descriptive month-to-month variability in *Klebsiella* CRE rates (range 13.5–32.0%)—which did not reach statistical significance in a formal trend test (Cochran–Armitage *p* = 0.13) but spanned a clinically relevant range—warrants continued surveillance.

These findings argue against a one-size-fits-all empirical protocol and support a stratified, locally guided approach: narrow-spectrum oral agents (nitrofurantoin, fosfomycin) for uncomplicated community UTI; early de-escalation away from fluoroquinolones and trimethoprim-sulfamethoxazole; and mechanism-aware, antibiogram-driven choices for *Klebsiella* and *Pseudomonas* in healthcare-associated settings, where carbapenems can no longer be assumed active.

The concentration of resistance in *Klebsiella* and the substantial month-to-month variability in CRE positivity together call for combined antimicrobial stewardship and infection control measures and for pairing phenotypic surveillance with molecular characterization to clarify the local NDM/OXA-48/KPC balance. Multi-center prospective studies, linkage to clinical outcomes (mortality, length of stay, treatment failure), and genomic tracking of resistant clones would extend the present descriptive picture.

By providing a comprehensive multi-organism antibiogram from a defined tertiary catchment, this study helps to close a real gap in Romanian urinary surveillance. It can inform both local protocols and the national debate on empirical UTI therapy in a country at the steep end of the European resistance gradient.

## Figures and Tables

**Table 1:** Species distribution of urinary isolates (*n* = 3348).

Organism	Isolates	% of Total
*Escherichia coli*	1356	40.5
*Enterococcus* spp.	742	22.2
*Klebsiella* spp.	632	18.9
*Proteus* spp.	203	6.1
*Pseudomonas* spp.	137	4.1
*Streptococcus* spp.	114	3.4
*Morganella morganii*	49	1.5
*Staphylococcus aureus*	47	1.4
*Acinetobacter* spp.	21	0.6
*Serratia* spp.	15	0.4
*Providencia* spp.	12	0.4
*Staphylococcus saprophyticus*	12	0.4
Other	8	0.2
Total	3348	100

**Table 2:** Overall burden of resistance phenotypes (*n* = 2331; first isolate per patient). Percentages are of all first isolates; putative ESBL is additionally expressed among *E. coli*, *Klebsiella* and *Proteus* (16.7%) and carbapenem resistance among *Enterobacterales* (3.9%). XDR and PDR were not classified (Methods).

Phenotype	Isolates	% of Total
MDR	357	15.3
Putative ESBL	263	11.3
CRE	63	2.7
VRE	1	0.04

**Table 3:** Resistance phenotypes by organism (% within each organism). The “—” denotes “not applicable/not tested”.

Organism	Putative ESBL	Carbapenem-R	MDR	VRE
*Escherichia coli* (*n* = 1059)	15.4	0.1	15.0	—
*Klebsiella* spp. (*n* = 395)	22.8	14.7	36.2	—
*Proteus* spp. (*n* = 123)	10.3	0.0	11.4	—
*Pseudomonas* spp. (*n* = 52)	—	48.1	36.5	—
*Enterococcus* spp.	—	—	1.1	0.2

**Table 4:** Antimicrobial resistance in *Escherichia coli* (% among tested isolates).

Antibiotic	Resistant/Tested	%R
Ertapenem	1/1059	0.1
Nitrofurantoin	13/1055	1.2
Fosfomycin	2/163	1.2
Amikacin	58/1059	5.5
Piperacillin-tazobactam	109/1057	10.3
Ceftriaxone/Ceftazidime/Cefotaxime	164/1059	15.5
Amoxicillin-clavulanate	249/1059	23.5
Levofloxacin	257/1059	24.3
Trimethoprim-sulfamethoxazole	333/1057	31.5

**Table 5:** Antimicrobial resistance in *Klebsiella* spp. (% among tested isolates).

Antibiotic	Resistant/Tested	%R
Amikacin	51/395	12.9
Ertapenem	58/393	14.8
Levofloxacin	95/394	24.1
Piperacillin–tazobactam	131/395	33.2
Trimethoprim–sulfamethoxazole	142/395	35.9
Ceftriaxone/Ceftazidime/Cefotaxime	143/395	36.2
Nitrofurantoin	159/391	40.7
Amoxicillin–clavulanate	211/395	53.4

**Table 6:** Antimicrobial resistance in *Enterococcus* spp. (% among tested isolates).

Antibiotic	Resistant/tested	%R
Vancomycin	1/512	0.2
Linezolid	1/520	0.2
Fosfomycin	2/291	0.7
Nitrofurantoin	16/517	3.1
Ampicillin	18/519	3.5
Penicillin	82/515	15.9
Levofloxacin	143/521	27.4

**Table 7:** Antimicrobial resistance in urease-positive organisms (% among tested isolates).

Antibiotic	Proteus (*n* = 123)	Morganella (*n* = 27)	Providencia (*n* = 4)
Amoxicillin–clavulanate	17.1	100	75.0
Piperacillin–tazobactam	4.1	11.1	25.0
Ceftazidime	8.9	14.8	50.0
Ertapenem	0.0	3.7	25.0
Amikacin	1.6	3.7	50.0
Trimethoprim–sulfamethoxazole	47.1	44.4	50.0

**Table 8:** Antimicrobial resistance in *Pseudomonas* spp. (% among tested isolates).

Antibiotic	Resistant/Tested	%R
Piperacillin–tazobactam	11/52	21.2
Amikacin	12/52	23.1
Ceftazidime	16/52	30.8
Imipenem	18/52	34.6
Levofloxacin	17/52	32.7
Cefepime	21/52	40.4
Meropenem	23/52	44.2

**Table 9:** Monthly isolate volume and resistance. *Klebsiella* spp. CRE percentages are of monthly *Klebsiella* isolates (denominators shown); other percentages are of the monthly isolate total. Resistance phenotypes use the revised definitions.

Month	Isolates	MDR%	CRE% (All)	Klebsiella CRE%
Feb. 2025	248	19.0	3.6	17.8 (8/45)
Mar. 2025	260	18.1	3.1	16.7 (7/42)
Apr. 2025	200	11.5	2.5	13.5 (5/37)
May. 2025	271	15.5	2.6	15.2 (7/46)
Jun. 2025	255	6.5	4.3	23.4 (11/47)
Jul. 2025	324	18.5	4.6	20.0 (15/75)
Aug. 2025	198	18.2	4.0	32.0 (8/25)
Sep. 2025	292	21.6	5.8	19.2 (14/73)
Oct. 2025	362	20.7	3.9	14.5 (12/83)
Nov. 2025	334	16.5	5.1	24.2 (16/66)
Dec. 2025	189	18.0	2.6	17.9 (5/28)
Jan. 2026	253	18.6	4.3	27.5 (11/40)
Feb. 2026	162	18.5	4.9	28.0 (7/25)

**Table 10:** Sensitivity analysis: principal resistance estimates at the isolate level (*n* = 3348) and restricted to the first isolate per unique patient (*n* = 2331).

Metric	Isolate-Level (*n* = 3348)	First-Isolate (*n* = 2331)	Δpp
MDR overall	18.0%	15.3%	−2.7
Putative ESBL overall	11.8%	11.3%	−0.5
CRE overall (all isolates)	3.9%	2.7%	−1.3
CRE (*Enterobacterales*)	5.9%	3.9%	−1.9
*E. coli* MDR	16.4% (222/1356)	15.0% (159/1059)	−1.4
*E. coli* putative ESBL	17.1% (232/1356)	15.4% (163/1059)	−1.7
*Klebsiella* MDR	41.1% (260/632)	36.2% (143/395)	−4.9
*Klebsiella* putative ESBL	22.6% (143/632)	22.8% (90/395)	+0.2
*Klebsiella* CRE	19.9% (126/632)	14.7% (58/395)	−5.2
*Pseudomonas* MDR	41.6% (57/137)	36.5% (19/52)	−5.1
*Pseudomonas* CR	51.1% (70/137)	48.1% (25/52)	−3.0
*Proteus* MDR	12.3% (25/203)	11.4% (14/123)	−0.9

## Data Availability

An anonymized dataset is available from the corresponding author on reasonable request, subject to institutional ethics committee approval, in accordance with *Microorganisms* policy.

## Supplementary Materials

The following supporting information can be downloaded at: https://www.mdpi.com/article/10.3390/microorganisms14071491/s1, Figure S1: Enzymatic hydrolysis of β-lactams by ESBLs and CRE; Figure S2: Urease-driven alkalinization and struvite/carbonate–apatite crystal formation; Table S1: Overall resistance phenotypes, full isolate-level dataset (*n* = 3348); Table S2: Antimicrobial resistance in *Escherichia coli* (isolate level); Table S3: Antimicrobial resistance in *Klebsiella* spp. (isolate level); Table S4: Antimicrobial resistance in *Enterococcus* spp. (isolate level); Table S5: Antimicrobial resistance in *Pseudomonas* spp. (isolate level).
