# Supplementary material for: The Resistance Landscape of Uropathogens in a Romanian Tertiary Center: A 13-Month Single-Center Study with Focus on Klebsiella and Urease-Positive Organisms
Source: Microorganisms. 2026 Jul 8;14(7):1491. doi: 10.3390/microorganisms14071491 (PMC13413781; doi:10.3390/microorganisms14071491)
Supplement: Supplementary file 1 [file microorganisms-14-01491-s001.zip › microorganisms-4375560-supplementary.pdf]

Supplementary Material

Resistance landscape of uropathogens at a Romanian tertiary centre (Manuscript ID microorganisms-4375560)

The main text reports first-isolate-per-patient estimates (primary analysis, n = 2331). The tables below report the full isolate-level dataset (secondary analysis, n = 3348), in which recurrent cultures are counted repeatedly; estimates are therefore systematically higher than the primary first-isolate values. All phenotypes use the revised definitions (MDR = resistance in ≥ 3 antimicrobial classes; putative ESBL = resistance to ≥ 1 of ceftazidime / ceftriaxone / cefotaxime with carbapenem susceptibility, restricted to Escherichia coli, Klebsiella spp. and Proteus mirabilis; CRE = carbapenem-resistant Enterobacterales by ertapenem-primary screening). Agent-level percentages use tested isolates as the denominator.

Table S1. Overall resistance phenotypes, full isolate-level dataset (n = 3348).

Phenotype	Resistant / tested	% R
Multidrug-resistant (MDR)	601 / 3348	18.0
Putative ESBL (*E. coli*, *Klebsiella*, *Proteus*)	396 / 2191	18.1
Carbapenem-resistant *Enterobacterales* (CRE)	135 / 2275	5.9
Vancomycin-resistant enterococci (VRE)	1 / 731	0.1

Putative ESBL corresponds to 11.8% and CRE to 4.0% when expressed over all 3348 isolates. VRE denominator is enterococci tested for vancomycin.

Table S2. Antimicrobial resistance in *Escherichia coli*, isolate-level (n = 1356; % among tested).

Antibiotic	Resistant / tested	% R
Amoxicillin-clavulanate	340 / 1356	25.1
Piperacillin-tazobactam	142 / 1354	10.5
Ceftazidime / ceftriaxone	234 / 1356	17.3
Ertapenem	2 / 1356	0.1
Levofloxacin	359 / 1356	26.5
Amikacin	71 / 1355	5.2
Nitrofurantoin	20 / 1345	1.5
Fosfomycin	3 / 193	1.6
Trimethoprim-sulfamethoxazole	458 / 1354	33.8

Table S3. Antimicrobial resistance in *Klebsiella spp.*, isolate-level (n = 632; % among tested).

Antibiotic	Resistant / tested	% R
Amoxicillin-clavulanate	362 / 632	57.3
Piperacillin-tazobactam	229 / 631	36.3
Ceftazidime / ceftriaxone	263 / 632	41.6
Ertapenem	126 / 628	20.1
Levofloxacin	186 / 631	29.5
Amikacin	116 / 628	18.5
Nitrofurantoin	272 / 625	43.5
Trimethoprim-sulfamethoxazole	249 / 629	39.6

Table S4. Antimicrobial resistance in *Enterococcus spp.*, isolate-level (n = 742; % among tested).

Antibiotic	Resistant / tested	% R
Ampicillin	29 / 740	3.9
Penicillin	142 / 735	19.3
Levofloxacin	237 / 742	31.9
Nitrofurantoin	26 / 736	3.5
Fosfomycin	7 / 373	1.9
Vancomycin	1 / 731	0.1
Linezolid	2 / 741	0.3

Table S5. Antimicrobial resistance in *Pseudomonas spp.*, isolate-level (n = 137; % among tested).

Antibiotic	Resistant / tested	% R
Piperacillin-tazobactam	21 / 136	15.4
Amikacin	39 / 137	28.5
Ceftazidime	41 / 136	30.1
Imipenem	50 / 136	36.8
Levofloxacin	51 / 137	37.2
Cefepime	55 / 136	40.4
Meropenem	68 / 136	50.0

Figure S1. Enzymatic hydrolysis of the β-lactam ring and the phenotypic definitions (putative ESBL and carbapenem resistance) applied in this study. The phenotypic approach identifies resistance behaviour and cannot distinguish specific enzyme classes.

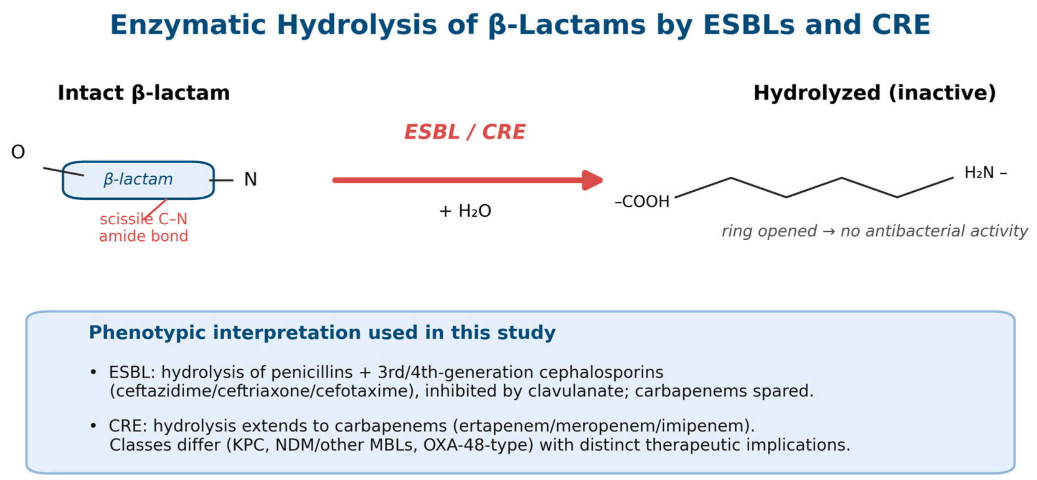

Figure S2. Urease-driven alkalinization and struvite / carbonate-apatite crystal formation — the mechanistic basis linking urease-positive uropathogens (*Proteus spp.*, *Morganella morganii*, *Providencia spp.*) to infection (struvite) stones.

Urease-Driven Alkalinization and Struvite Crystal Formation

Step 1 — Urea hydrolysis (bacterial urease)

$$(NH_2)_2CO + 2\,H_2O \xrightarrow{\text{urease}} 2\,NH_3 + CO_2$$

Step 2 — Ammonia raises urinary pH

$$NH_3 + H_2O \longrightarrow NH_4^+ + OH^- \quad \text{(pH ↑ → 7.5–9)}$$

Step 3 — Crystal precipitation at alkaline pH

$$Mg^{2+} + NH_4^+ + PO_4^{3-} + 6\,H_2O \longrightarrow MgNH_4PO_4{\cdot}6H_2O \quad \text{(struvite)}$$

$$10\,Ca^{2+} + 6\,PO_4^{3-} + 2\,OH^- \longrightarrow Ca_{10}(PO_4)_6(OH)_2 \quad \text{(carbonate-apatite)}$$

Clinical consequence

Crystalline biofilms encase bacteria, shielding them from antibiotics and host defenses, obstruct catheters, and seed infection (struvite/staghorn) stones — a self-perpetuating cycle of obstruction, recurrence and treatment failure. Up to ~75% of staghorn stones are struvite/carbonate-apatite.

Urease-producing uropathogens: Proteus spp. (foremost), Morganella morganii, Providencia spp., and some Klebsiella and staphylococci.
